# Comprehensive Evaluation of Sporadic Late-Onset Cerebellar Ataxias: Clinical Presentation, Diagnostic Challenges, and Treatment Outcomes

**DOI:** 10.7759/cureus.62667

**Published:** 2024-06-19

**Authors:** Intissar Barbouch, Amina Ali Kako, Yassine Mebrouk

**Affiliations:** 1 Department of Neurology, Mohammed Vl University Hospital, Faculty of Medicine and Pharmacy, Mohammed First University, Oujda, MAR

**Keywords:** outcome, dysmetria, ataxia, cerebral, sporadic

## Abstract

Sporadic late-onset cerebellar ataxias (SLOCA) present a diagnostic challenge due to their heterogeneous etiologies and complex clinical manifestations. This retrospective study aimed to conduct a comprehensive evaluation of six male patients diagnosed with SLOCA, with a mean age of 55 years and an average symptom onset at 47 years. All patients presented with gait and balance disturbances, with additional sensory abnormalities observed in two cases. Neurological examinations revealed varied cerebellar syndromes, including static and static-kinetic presentations, accompanied by peripheral neurogenic syndromes in some instances. Brain MRI findings showed cerebellar atrophy, predominantly involving the vermis, in a subset of patients. Biochemical and serological investigations yielded mostly unremarkable results, although two patients exhibited significant vitamin E deficiency and anti-Hu antibodies (anti-neuronal nuclear antibody type 1). Electromyography confirmed sensory axonal neuropathy in those with peripheral neurogenic syndromes. Treatment with TOCO 500 mg (Vitamin E) was administered to four patients, with follow-up indicating stable disease progression in two cases. This study underscores the complexity of SLOCA and the need for a multidisciplinary approach to diagnosis and management. Further research is warranted to elucidate the underlying mechanisms and improve clinical outcomes for affected individuals.

## Introduction

Sporadic late-onset cerebellar ataxias (SLOCA) represent a complex and heterogeneous group of neurodegenerative disorders characterized by progressive cerebellar dysfunction [1]. These conditions typically manifest with gait disturbances, balance problems, and, in some cases, additional neurological deficits. Despite advances in medical research, the etiologies of SLOCA remain varied and often elusive, necessitating extensive and multifaceted diagnostic approaches [2]. Currently, there is no standardized protocol for the diagnosis and treatment of SLOCA, which poses significant challenges for clinicians [2,3]. The primary objective of this study is to conduct a comprehensive evaluation of SLOCA, focusing on their clinical presentations, paraclinical investigations, and disease progression. By identifying potential etiological factors and proposing structured diagnostic and therapeutic strategies, this study aims to enhance the understanding and management of SLOCA.

## Materials and methods

We conducted a comprehensive retrospective analysis of six patient records, all of whom were diagnosed with SLOCA, characterized by an age of onset exceeding 40 years. The cohort was exclusively composed of male patients.

Patients were selected based on a clinical diagnosis of cerebellar ataxia confirmed through neurological assessments and imaging studies. Inclusion criteria required a definitive diagnosis of SLOCA, and exclusion criteria ruled out hereditary and familial forms of the disease.

The diagnostic workup encompassed a multifaceted approach. A detailed assessment of cerebellar function was conducted for each patient, including evaluations of ataxia using standardized scales, tests for dysmetria (finger-to-nose and heel-to-shin tests), and other cerebellar signs such as nystagmus and speech disturbances. Brain MRI scans were performed on all patients using the same device, a 3 Tesla MRI scanner. These scans were meticulously reviewed for signs of cerebellar atrophy, with a specific focus on the vermis. Imaging protocols included T1-weighted, T2-weighted, and FLAIR sequences to provide a comprehensive assessment of cerebellar and surrounding brain structures.

A series of laboratory tests were conducted to identify potential metabolic and infectious etiologies. These included comprehensive metabolic panels, liver and renal function tests, and serum levels of vitamins B9 (folate), B12 (cobalamin), and E (tocopherol) to detect deficiencies. Viral serologies were performed to screen for hepatitis B virus (HBV), HIV, and syphilis. A CSF analysis was conducted via lumbar puncture to identify abnormalities indicative of an infectious or inflammatory process. Electromyography (EMG) was used to assess peripheral nerve function and muscle response, providing additional data on neuromuscular involvement. Despite the absence of a family history in some cases, genetic evaluations were deemed essential.

## Results

All six patients were male, with a mean age of 55 years (SD = 4.2) and an average age of symptom onset at 47 years (SD = 3.5). The median age was 55 years (IQR = 52-58), and the median age of symptom onset was 47 years (IQR = 44-50). They all presented with gait and balance disturbances, except for two patients who also exhibited sensory disturbances, such as tingling.

**Figure 1:**
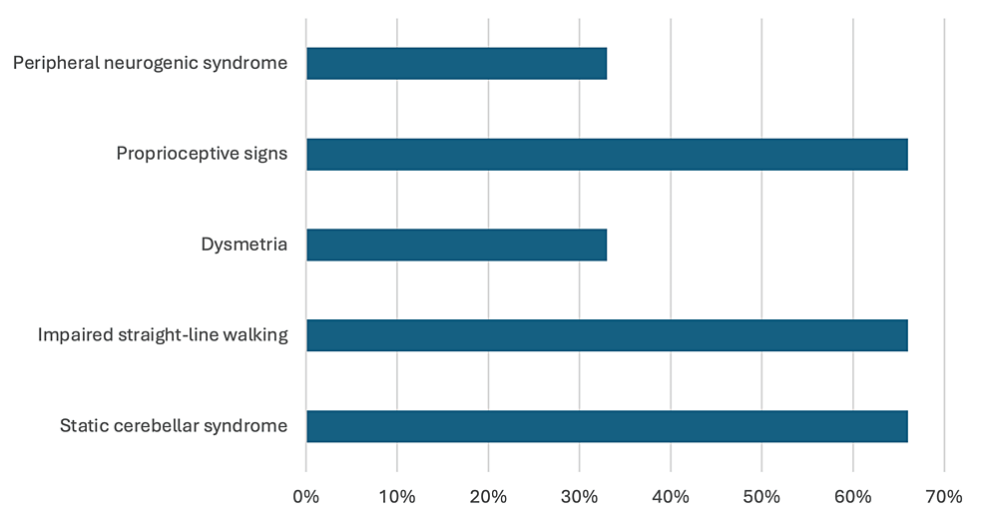
Clinical presentation of the patients

Neurological examinations revealed a static cerebellar syndrome in four patients, characterized by an unsteady gait, difficulty in turning, and impaired straight-line walking, which worsened with eye closure. Two patients had static-kinetic cerebellar syndrome, exhibiting dysmetria during the finger-to-nose test, along with peripheral neurogenic syndrome. Additionally, one patient showed athetotic movements in both hands (Figure 1).

**Figure 2:**
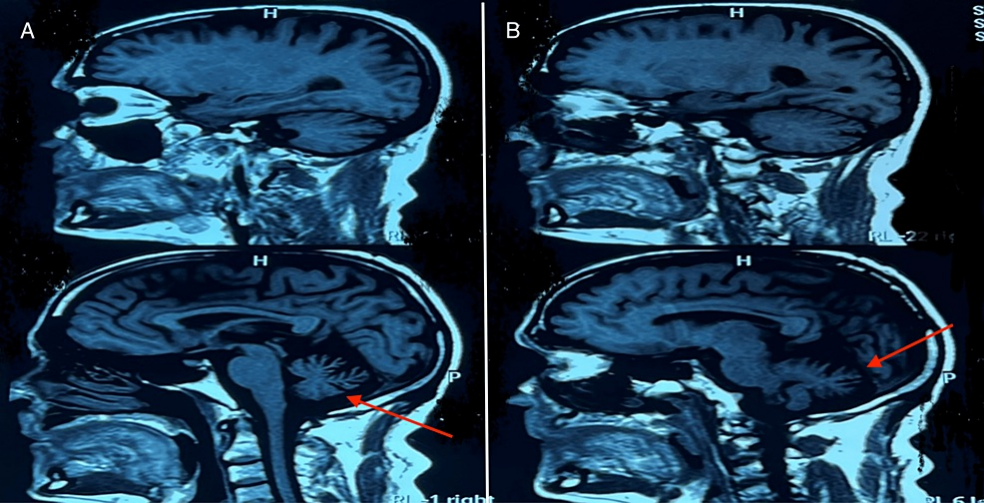
Brain MRI findings showed cerebellar atrophy with a vermian predominance (red arrows) in two patients (A and B)

Brain MRI findings showed cerebellar atrophy with vermian predominance in two patients (Figure 2), while the other four had normal MRI results. Biochemical and serological tests, including standard biological assessments, vitamin levels (B9, B12, and VitE), thyroid function tests, viral serologies (HBV, HIV, and syphilis), CSF studies, tumor markers, and anti-neuronal antibodies, were mostly unremarkable. However, one patient had a significant vitamin E deficiency (11 ng/l), and another tested positive for anti-Hu antibodies.

EMG revealed moderate sensory axonal neuropathy in the two patients with peripheral neurogenic syndrome. Four patients were treated with TOCO 500 mg (3,000 mg/day), while the other two did not receive any treatment.

Follow-up data showed that two patients were seen after two years, with their condition remaining stable. The other patients were lost to follow-up.

## Discussion

SLOCA present a significant diagnostic challenge due to their heterogeneous etiologies and overlapping clinical features [4]. Our findings underscore the necessity for a thorough and systematic diagnostic approach, incorporating radiological, genetic, metabolic, paraneoplastic, inflammatory, and symptomatic evaluations. This comprehensive strategy is crucial for accurately identifying the underlying cause of SLOCA and guiding appropriate management.

In our study, multisystem atrophy (MSA) emerged as the most frequently identified cause of SLOCA, as four of our patients had MSA, highlighting its prevalence among these cases in comparison to the literature [5,6]. This finding aligns with the existing literature, which indicates that MSA is a common etiology in late-onset cerebellar ataxia. However, the complexity of SLOCA necessitates advanced diagnostic techniques, including genetic analyses, which were beyond the scope of our current study [7]. The absence of genetic testing is a limitation that may have precluded the identification of hereditary ataxias in our cohort.

Supporting our findings, a prospective study from the University Hospital of Strasbourg, involving 80 patients over two years, reported that 65% of the patients received a definitive diagnosis, with MSA being the predominant cause [8]. This study further demonstrated the diverse etiologies of SLOCA, with 35% of patients having conditions such as autosomal dominant or recessive hereditary genetic disorders, autoimmune conditions, and toxic exposures. This variability underscores the complexity of SLOCA and the need for personalized diagnostic pathways [8].

The identification of specific etiologies in SLOCA often requires advanced diagnostic techniques. For instance, genetic analyses can reveal hereditary ataxias, which may present similarly to sporadic cases [9,10]. Furthermore, paraneoplastic syndromes, which can mimic other forms of ataxia, require extensive serological testing to identify underlying malignancies. Inflammatory and autoimmune causes, such as gluten ataxia or autoimmune cerebellitis, necessitate a range of immunological tests to confirm diagnosis [9-11].

Metabolic causes of SLOCA, such as deficiencies in vitamins B9, B12, and E, highlight the importance of thorough biochemical assessments. These deficiencies are often treatable, and early identification can significantly alter disease progression and patient outcomes. Additionally, toxic exposures, although less common, must be considered, particularly in patients with relevant occupational or environmental histories [12-15].

Our study highlights the importance of a multidisciplinary approach to diagnosing SLOCA. Collaboration between neurologists, geneticists, radiologists, and other specialists is essential to navigate the complex diagnostic landscape of these disorders. Future research should aim to integrate advanced diagnostic modalities, including next-generation sequencing and comprehensive metabolic panels, to enhance diagnostic accuracy and identify novel etiological factors.

While our study emphasizes the prevalence of MSA in SLOCA, it also illustrates the necessity for a broad and integrated diagnostic approach. The diversity of potential etiologies requires that clinicians remain vigilant and exhaustive in their diagnostic evaluations, ensuring that patients receive accurate diagnoses and appropriate, targeted treatments.

Due to logistical constraints, genetic studies could not be performed. This limitation restricts our ability to fully explore genetic contributions to the etiology of late-onset cerebellar ataxia within our cohort. By integrating clinical, imaging, biochemical, and serological data, we aimed to provide a thorough diagnostic evaluation of SLOCA in our patient sample. Future studies incorporating genetic testing are warranted to enhance our understanding of the underlying mechanisms contributing to this condition.

## Conclusions

SLOCA predominantly affect men over the age of 40 and are characterized by significant gait and balance disturbances. Comprehensive and systematic investigations are crucial for identifying the diverse etiologies of these conditions. Despite the extensive diagnostic efforts, a well-defined and standardized diagnostic strategy remains elusive. Our study highlights the need for further research to develop more effective diagnostic and therapeutic protocols for SLOCA. An enhanced understanding of the underlying causes will ultimately improve patient outcomes and provide clearer guidance for clinicians managing these complex disorders.
